# A retrospective clinical analysis of inappropriate Xiangdan Injection use: clinical risk profiling and computational hypothesis generation

**DOI:** 10.3389/fphar.2026.1742792

**Published:** 2026-02-11

**Authors:** Ji Zeng, Yougang Wei, Jinke Zhang, Yiming Xu

**Affiliations:** 1 Clinical Pharmacy Department, Department of Pharmacy, Ma’anshan City Hospital of Traditional Chinese Medicine, Ma’anshan, Anhui, China; 2 Oncology Department, Ma’anshan City Hospital of Traditional Chinese Medicine, Ma’anshan, Anhui, China

**Keywords:** Xiangdan Injection, inappropriate drug use, retrospective study, clinical risk assessment, network pharmacology, molecular docking, adverse drug reaction

## Abstract

**Purpose:**

Xiangdan Injection, a traditional Chinese medicine preparation, is extensively utilized in clinical settings. Nevertheless, frequent instances of inappropriate administration pose significant safety concerns. The clinical findings were supplemented with *in silico* analyses (network pharmacology and molecular docking) to generate hypotheses regarding potential bioactive constituents and molecular pathways.

**Methods:**

A retrospective analysis was performed on 100 patients who received Xiangdan Injection at Ma’anshan Hospital of Traditional Chinese Medicine. A focused review was undertaken to assess the rationale for using Xiangdan Injection in these 100 patients. Furthermore, as an exploratory supplement, we employed network pharmacology and molecular docking methods, aiming to generate hypotheses regarding the potential basis of their biological activities.

**Results:**

An analysis of 100 clinical cases identified four primary patterns of irrational drug use: unclear indications (67.8%), inappropriate solvent selection (16.1%), repeated medication (11.3%), and prolonged treatment duration (4.8%). Exploratory network pharmacology suggested that key metabolites (e.g., luteolin) may target proteins such as AKT1 and PTGS2, and potentially associate with pathways like PI3K/AKT. Molecular docking indicated a potential binding affinity between luteolin and PTGS2, offering a preliminary hypothesis for its pharmacological profile. Furthermore, these metabolites were linked to liver-related processes, suggesting a need for vigilance regarding hepatotoxicity with prolonged use.

**Conclusion:**

This study identified the main risk patterns of the irrational clinical use of Xiangdan Injection. The computational part of this study put forward hypotheses that need to be verified by future experiments. We also proposed targeted suggestions for improving the safety of clinical medication use.

## Introduction

Xiangdan Injection is a traditional Chinese medicine formulation derived from *Salvia miltiorrhiza* and *Dalbergia odorifera*, recognized for its vasodilatory effects and its capacity to enhance coronary artery blood flow. Clinically, it is primarily utilized to treat cardiovascular diseases, such as angina pectoris and myocardial infarction (Shen et al., 2010). With the expansion of its clinical use, instances of inappropriate administration have increased, along with a rise in reported adverse reactions. Recent studies have reported a range of adverse reactions associated with Xiangdan Injection, such as allergic skin reactions, anaphylactic shock, laryngeal edema, severe diarrhea, and hepatorenal impairment (Huang et al., 2021).

The safety assessment of traditional Chinese medicine (TCM) injections has attracted considerable attention from the scientific community. Recent advancements have established standardized methods for evaluating allergic reactions and screening allergenic components in TCM injections, providing innovative approaches for safety research (Huang et al., 2021). Although previous studies have investigated adverse reactions to Xiangdan injection, systematic analyses of irrational clinical usage patterns and their underlying mechanisms are still limited (Wang et al., 2023). Most previous research has concentrated on incident reporting or descriptive epidemiology, resulting in a gap in understanding how specific improper medication practices, such as inappropriate solvent selection or prolonged duration, may mechanistically contribute to risk (Huang et al., 2021; Dai et al., 2024; Gao et al., 2024). Therefore, this study was designed to systematically evaluate the clinical risk profiles associated with irrational Xiangdan injection use through a 100-case retrospective analysis. This clinical assessment was supplemented with a hypothesis-generating, *in silico* exploration employing network pharmacology and molecular docking to identify preliminary links between active constituents and pathways for future validation (Liu et al., 2023). Collectively, our findings aim to inform safer clinical practice.

## Materials and methods

### Study material: Xiangdan injection

Xiangdan injection is a standardized, sterile aqueous preparation approved for clinical use in China by National Medical Products Administration (NMPA). This study analyzed the clinical application of this commercially available, approved formulation. The injection is produced from the following botanically validated source materials: *S. miltiorrhiza* Bunge [Lamiaceae] and *Dalbergia odorifera* T.C. Chen [Fabaceae].

Its quality control, manufacturing process, and specifications are defined in the official monograph of the Chinese Pharmacopeia (2020 Edition). For the subsequent network pharmacology analysis, the chemical constituents of these two herbs were retrieved from public databases.

### Data collection and statistical analysis

**FIGURE 1:**
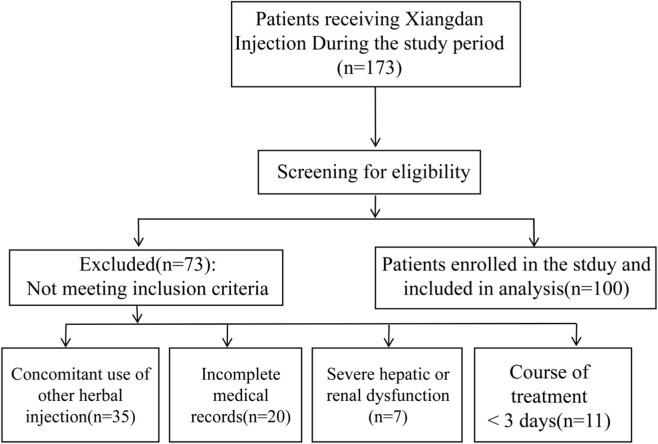
Flowchart of patient selection. This flowchart meticulously depicts the entire screening process, commencing from the initial identification of all patients treated with Xiangdan injection (n = 173) and culminating in the final cases incorporated into the analysis (n = 100). It also details the specific reasons and quantities of excluded cases.

We conducted a retrospective analysis of 100 inpatient medical records of patients treated with the aforementioned Xiangdan Injection at Ma’anshan Hospital of Traditional Chinese Medicine from October 2023 to September 2025.Initially, 173 medical records of patients who received Xiangdan Injection were identified. After applying the predefined inclusion and exclusion criteria, 100 cases were included for analysis. The detailed patient screening process is illustrated in Figure 1. The inclusion criteria were: (1) age ≥18 years; (2) treatment with Xiangdan Injection for at least 3 days (chosen as it represents a complete short-term treatment cycle, facilitating a more meaningful assessment of prescription rationale and potential adverse effects); and (3) complete medical records. The exclusion criteria included: (1) concurrent use of other Chinese herbal injections; (2) pregnancy or lactation; and (3) severe hepatic or renal dysfunction (Child-Pugh grade C or eGFR <30 mL/min/1.73 m^2^).

Criteria for inappropriate medication use were established through a consensus process by a dedicated expert review panel of clinical pharmacists and physicians. The panel derived the criteria (Jiang, 2023) based on a synthesis of: (1) the “Principles for Clinical Use of Herbal Injections,” (2) the official prescribing information for Xiangdan Injection, and (3) relevant clinical guidelines. To ensure consistency and validity, the panel employed a modified Delphi method (Humphrey-Murto and de Wit, 2019), discussing and refining the criteria over several rounds to achieve a high level of inter-reviewer agreement. Inappropriate use was categorized as: (1) absence of a documented diagnosis corresponding to the approved indications for Xiangdan Injection; (2) excessively prolonged therapy duration (≥14 consecutive days); (3) inappropriate solvent selection (i.e., using solvents other than the recommended 5% glucose injection); and (4) duplicate therapy involving concurrent use of other herbal medicines with similar Huoxue Huayu (blood circulation-promoting and stasis-resolving) effects.

To mitigate potential biases, data extraction was conducted by two independent reviewers utilizing a standardized form. Discrepancies or instances of missing critical data (e.g., unspecified solvents, missing diagnosis codes) were addressed by re-examining the original records and, if necessary, through adjudication by a third senior panel member. Potential confounding variables, such as patient age, primary diagnosis, comorbidities, and concomitant non-herbal medications, were systematically gathered for descriptive analysis. This study received review and approval from the Ethics Committee of Ma’anshan Hospital of Traditional Chinese Medicine (Approval Reference Number: (2024) Lun Shen Yan No. (25)). The requirement for informed consent was waived because of the retrospective nature of the analysis.

Statistical analyses were performed using GraphPad Prism (version 10.4). Categorical variables are presented as frequencies and percentages (n, %).

### Collection of metabolites and associated targets

To construct a metabolite-target network, the major metabolites of *S. miltiorrhiza* and *Dalbergia odorifera* (the botanical sources of Xiangdan Injection) were sourced from TCMSP (Au-aff, 2014). To refine this list to metabolites with higher potential for biological activity, a pharmacokinetic filter (OB ≥ 30% and DL ≥ 0.18) was applied (Zhao et al., 2023). The potential targets of these filtered metabolites were then predicted. A consolidated, non-redundant target list was generated after removing duplicates for further analysis.

### Integration of disease targets and network analysis

Target related to cancer-related fatigue, liver injury, and allergies were identified using GeneCards (Gil, 2016) (https://www.genecards.org/), OMIM (Joanna and Ada, 2017) (https://www.omim.org/), and TTD (Ying, 2023) (https://db.idrblab.net/ttd). The overlap between these disease targets and those predicted for Xiangdan Injection metabolites was identified via Venn analysis. The resulting common targets were used as the basis for constructing and topologically analyzing a protein-protein interaction network with Cytoscape.

### Construction of protein-protein interaction networks

The shared targets, identified from the intersection of metabolite-related and disease-related genes, were imported into STRING (Mehryary, 2024) (https://string-db.org) to construct a protein-protein interaction (PPI) network. The search was restricted to *Homo sapiens* with a medium confidence interaction score threshold (≥0.400). The resulting network was then imported into Cytoscape 3.8.2 (Li et al., 2022) for subsequent topological analysis. To identify core targets within the network, nodes were first filtered based on three centrality metrics (degree, betweenness centrality, and closeness centrality), retaining those exceeding the median value for each measure. The top 10 key targets were ultimately selected by ranking in descending order of degree centrality.

### GO and KEGG enrichment analysis

Gene Ontology (GO) functional annotation and Kyoto Encyclopedia of Genes and Genomes (KEGG) pathway enrichment analyses for the intersection targets were conducted using the Database for Annotation, Visualization, and Integrated Discovery (DAVID, version 6.8) (Glynn, 2003) (https://davidbioinformatics.nih.gov/). The analysis was conducted with the background species parameter explicitly set to” *H. sapiens*”. Enrichment results were filtered using a significance threshold of p < 0.05 and sorted by p-value in ascending order. For subsequent visualization and interpretation, the top 10 most significantly enriched GO terms (Biological Process) and the top 20 most significantly enriched KEGG pathways were selected (Lei, 2017).

### Molecular docking

Molecular docking was utilized to evaluate the binding affinity between bioactive compounds and their protein targets (Pinzi and Rastelli, 2019). The top five potential targets and their corresponding ligands were identified based on the highest rank scores obtained from the Maximal Clique Centrality (MCC) algorithm analysis of the protein-protein interaction network (Alamro et al., 2023). The three-dimensional structural files (in SDF format) for the five candidate compounds were obtained from the PubChem database. Before docking, ligand structures were prepared by minimizing their energy and assigning appropriate protonation states at a physiological pH of 7.4 using the Molecular Operating Environment (MOE) software (Farwa and Raza, 2022). The three-dimensional crystal structure of the primary target receptor (in PDB format) was obtained from the RCSB Protein Data Bank. The receptor structure was prepared by removing water molecules and co-crystallized ligands, adding polar hydrogen atoms, and assigning Kollman united-atom charges. Docking simulations were conducted using AutoDock Vina software (version 1.2.). The binding site was defined by constructing a grid box centered on the coordinates of the native ligand in the crystal structure, with dimensions of 20 × 20 × 20 Å and a grid point spacing of 0.375 Å. An exhaustiveness parameter of eight was established for the conformational search to balance computational accuracy and efficiency. For each ligand-receptor pair, the docking simulation was repeated three times, and the conformation with the most favorable (lowest) calculated binding energy (ΔG, kcal/mol) was chosen as the predicted binding pose. All binding energies were systematically documented for comparative analysis.

### Interpretation and limitations of silico predictions

It is important to note that the predictions from network pharmacology and molecular docking are hypothesis-generation in nature. Identified compounds, such as luteolin, are known to exhibit pan-assay interference properties in certain experimental contexts. Therefore, the interaction profiles predicted here represent canonical binding possibilities based on structural models and do not constitute definitive evidence of biological activity. These results require validation through orthogonal biochemical and functional assays.

## Results

### Patterns of inappropriate prescribing

An initial review of 173 prescriptions (detailed in Figure 1) identified 100 inpatient cases that met the inclusion criteria for analysis. The demographic and clinical characteristics of the final cohort are summarized as follows: the mean age was 68.2 years (±11.8), with 43% of the patients being male. The most common primary diagnoses were oncology-related conditions (38%), cardiovascular diseases (49%), and other disorders (13%).

Our analysis revealed an overall inappropriate usage rate of 62.0% for Xiangdan Injection (62 out of 100 cases). Table 1 presents the distribution of various identified categories of inappropriate practices. It is essential to note that the percentages for each category are calculated based on the total number of inappropriate use cases (n = 62). These percentages do not sum to 100% because individual cases may involve multiple types of inappropriate practices. The most prevalent category was administration without a clear, documented indication (42 cases, 67.8% of inappropriate cases), followed by the use of an unsuitable diluent (10 cases, 16.1%), therapeutic duplication (7 cases, 11.3%), and excessive treatment duration (3 cases, 4.8%).

**TABLE 1:** Profile of inappropriate Xiangdan injection Use(N = 62).

Category of inappropriate use	Cases, N	Proportion (%)
Lack of clear indication	42	67.7
Inappropriate solvent selection	10	16.1
Drug duplication	7	11.3
Prolonged therapy	3	4.8

Further analysis indicated that administration without a clear indication was common among oncology patients (21 out of 42, 50.0%), primarily for managing cancer-related fatigue. Among the three patients receiving prolonged therapy, mild elevation in liver function tests was noted in one case (33.3%). Additionally, the use of an inappropriate diluent was linked to dermal reactions (e.g., pruritus) in 10.0% of affected cases (1 out of 10).

### Clinical case analysis

To further clarify the clinical manifestations and potential risks associated with the primary categories of inappropriate use identified in this study, we conducted an in-depth analysis of three representative cases from our cohort. We performed a causality assessment for suspected adverse drug reactions (ADRs) using the Naranjo algorithm. The key clinical features, identified issues, and implications of each case are summarized in Table 2 and briefly described below.

**TABLE 2:** Summary of key clinical cases demonstrating inappropriate use of Xiangdan Injection.

Case	Clinical context	Identified inappropriateness	Key clinical implication
1	Prostate cancer with CRF(KPS = 40). Received 4-day course	Off-label use for CRF. Symptom improvement (KPS = 50)	Highlights prevalent off-label use. Warrants systematic research to establish evidence
2	Erysipelas patient. Received injection + oral salvia decoction for 20 days	Prolonged therapy (≥14 d) +therapeutic duplication. Asymptomatic ALT/AST elevation; Naranjo: Probable	Emphasizes hepatotoxicity risk from prolonged use. Necessitates duration limits
3	Breast cancer with hypercoagulability. Injection diluted in normal saline	Protocol deviation (diluent).Pruritus within 24h; resolved after stop. Naranjo: Probable	Confirms diluent safety-critical. Non-recommended solvents may increase hypersensitivity risk

### Case1: absence of a clear drug indication

Patient one was diagnosed with prostate cancer and cancer-related fatigue (CRF). He had a Karnofsky Performance Status (KPS) score of 40 upon presentation. His anticancer regimen consisted of apalutamide tablets and goserelin acetate implants. Upon admission, the patient displayed a pruritic rash. During hospitalization, the patient underwent a 4-day course of Xiangdan Injection, consisting of 20 mL diluted in 250 mL of 5% glucose solution. After adjunctive therapy, the patient reported reduced fatigue, and his KPS score increased to 50. According to Traditional Chinese Medicine (TCM) theory, blood stasis is a common pathogenic factor in cancer patients and may contribute to CRF. Xiangdan Injection, known for promoting blood circulation and resolving stasis, may alleviate fatigue and enhance quality of life in these cases. This case exemplifies the prevalent off-label use of Xiangdan Injection at our institution. Although robust evidence from randomized controlled trials is lacking, this clinical observation indicates potential utility in managing CRF and warrants further systematic investigation. This case underscores the prevalent off-label use of Xiangdan Injection for CRF management based on TCM theory, emphasizing the urgent need for robust clinical trials to establish its efficacy and safety.

### Case2: drug-induced liver injury due to prolonged therapy

Patient two was diagnosed with erysipelas (Traditional Chinese Medicine syndrome: Qi and Blood Deficiency Syndrome) and acute lower limb lymphangitis. The initial treatment involved intravenous amoxicillin-clavulanate for infection control, supplemented with Xiangdan Injection and a Chinese herbal decoction aimed at promoting Qi and blood circulation, which included Salvia miltiorrhiza (15 g) and Carthamus tinctorius (15 g). After 3 days, the antibiotic therapy was escalated to linezolid due to an inadequate clinical response. The patient had a history of multiple cerebral infarctions and was receiving long-term clopidogrel therapy. Upon admission, baseline liver function tests (LFTs) were within normal limits: alanine aminotransferase (ALT) 11 U/L, aspartate aminotransferase (AST) 9 U/L, and direct bilirubin (DBIL) 3.6 μmol/L. After 20 days continuous Xiangdan Injection therapy, the patient experienced fatigue and anorexia. Repeat liver function tests (LFTs) indicated elevated enzyme levels: ALT 33 U/L, AST 31 U/L, and DBIL 5.4 μmol/L. The Naranjo score for this event was classified as probable. Although these values remained below the threshold for clinical drug-induced liver injury, Xiangdan Injection was promptly discontinued as a precautionary measure. This case underscores the hepatotoxic potential associated with prolonged use and pharmacokinetic/pharmacodynamic duplication, emphasizing the need for strict duration limits, vigilant liver function monitoring, and avoidance of concomitant therapy with similar herbal components.

### Case3: anaphylactoid reaction due to inappropriate solvent selection

Patient three was diagnosed with breast cancer and exhibited an elevated D-dimer level of 765.7 μg/L, indicating a hypercoagulable state. Following Traditional Chinese Medicine principles related to blood stasis in cancer patients, Xiangdan Injection (20 mL diluted in 250 mL normal saline) was administered to enhance microcirculation and alleviate stasis. The following morning, the patient developed pruritus affecting the scalp and upper back. The symptoms gradually subsided and completely resolved within 24 h after discontinuation on day 3. The Naranjo score indicated a probable adverse reaction. This event underscores that the choice of diluent is a critical factor in ensuring safety. The use of non-recommended solvents may compromise product stability and elevate the risk of immediate hypersensitivity reactions, necessitating strict adherence to prescribing guidelines.

### Integration of metabolite and disease targets

**FIGURE 2:**
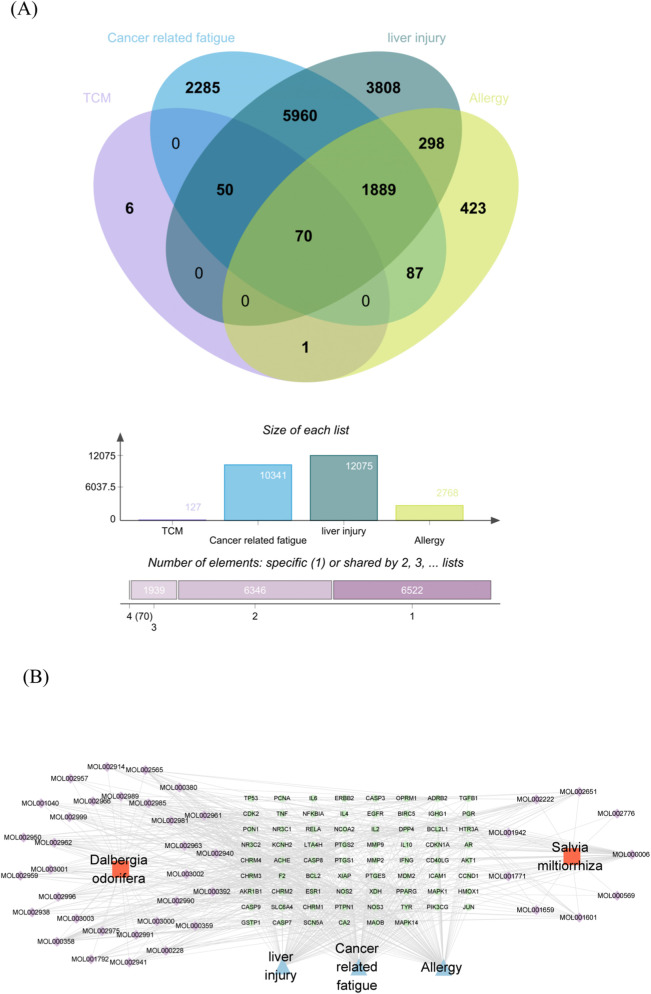
Identification and Network Representation of Shared Targets. **(A)** Venn diagram showing common targets between metabolite-related and disease-related gene sets (CRF, liver injury, and allergy). **(B)** Network visualization of the protein interactions among the shared targets from **(A)**.

**FIGURE 3:**
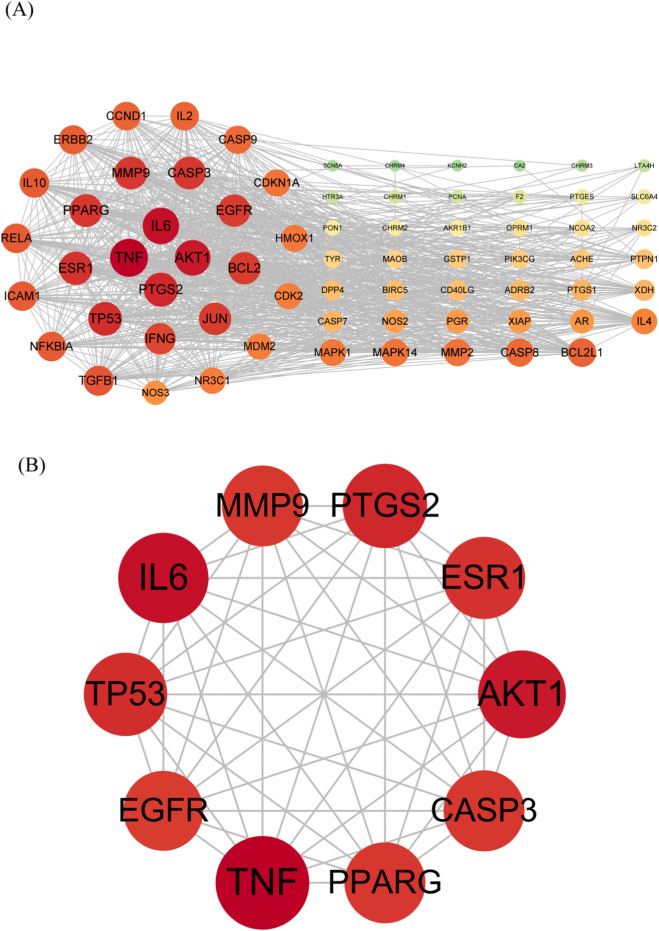
PPI network of the common targets for Xiangdan Injection and CRF in liver injury and allergy. **(A)** PPI network diagram plotted on the STRING network. **(B)** Ten key targets identified in the PPI network. Abbreviations: AKT1, AKT serine/threonine kinase 1; CASP3, caspase-3; EGFR, epidermal growth factor receptor; ESR1, estrogen receptor 1; IL-6, interleukin-6; MMP-9, matrix metallopeptidase-9; PPARG, peroxisome proliferator-activated receptor gamma; PTGS2, prostaglandin-endoperoxide synthase 2 (COX-2); TNF, tumor necrosis factor; TP53, tumor protein p53.

As an exploratory analysis to complement the clinical findings, network pharmacology was employed to identify potential targets and pathways. We identified a total of 127 major metabolites from the botanical drug components of Xiangdan Injection. Separate target sets for cancer-related fatigue (10,341 targets), liver injury (12,075 targets), and allergy (2,768 targets) were compiled. Comparative analysis identified 70 targets common to both the metabolite and disease target pools (work Table 3; Figure 2). A PPI network constructed from these shared targets revealed that several proteins, including TNF, IL6, AKT1, PTGS2, TP53, ESR1, MMP9, PPARG, CASP3, and EGFR, exhibited high connectivity and may represent hubs within the implicated biological network (Figure 3).

**TABLE 3:** Top 10 ranked metabolites and network-predicted associations.

TCM	Molecule name	MOL ID	Degree	Betweenness centrality	Closeness centrality
Salvia miltiorrhiza	Luteolin	MOL000006	41	0.066749	0.522936
Dalbergia odorifera	Beta-sitosterol	MOL000358	22	0.01977	0.44186
Dalbergia odorifera	(−)-Vestitol	MOL002961	21	0.011554	0.435115
Dalbergia odorifera	Formononetin	MOL000392	20	0.009478	0.425373
Dalbergia odorifera	4′,5′,7′-trimethyl-3-methoxyflavone	MOL002963	17	0.005701	0.419118
Dalbergia odorifera	Medicarpin	MOL002565	16	0.005942	0.416058
Dalbergia odorifera	Duartin	MOL002981	16	0.005864	0.419118
Dalbergia odorifera	Isoduartin	MOL002985	16	0.005446	0.419118
Salvia miltiorrhiza	1,2,5,6-Tetrahydrotanshinone	MOL001601	15	0.010161	0.419118
Dalbergia odorifera	4-Hydroxyhomopterocarpin	MOL002989	15	0.00461	0.416058

### GO function and KEGG pathway enrichment analysis

**FIGURE 4:**
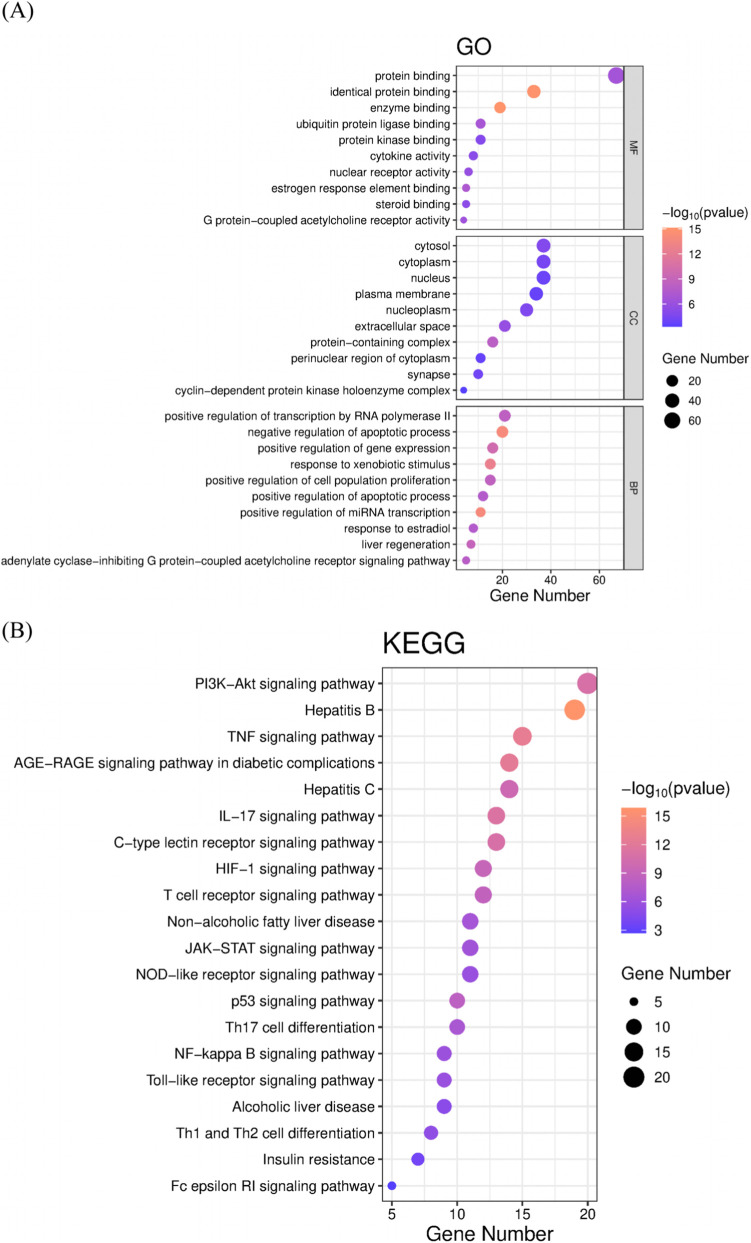
Top GO terms and KEGG pathways enriched by Xiangdan Injection in CRF, liver injury, and allergy. **(A)** Top GO terms enriched by Xiangdan Injection in CRF, liver injury, and allergy. The x-axis indicates the number of enriched genes, and the color of the dot represents the p-value for the corresponding term. **(B)** Top KEGG pathways enriched by Xiangdan Injection in CRF, liver injury, and allergy. A larger dot indicates a higher number of enriched genes in that pathway, and a dot with a darker red color indicates a smaller p-value. Abbreviations: HIF-1: hypoxia-inducible factor-1 signaling pathway; IL-17: interleukin-17 signaling pathway; JAK-STAT: Janus kinase-signal transducer and activator of transcription signaling pathway; NOD: nucleotide-binding oligomerization domain-like receptor signaling pathway; PI3K-Akt: phosphatidylinositol 3-kinase–protein kinase B signaling pathway; TNF: tumor necrosis factor signaling pathway.

GO and KEGG enrichment analyses identified 415 biological processes (BP), 54 cellular components (CC), 84 molecular functions (MF), and 143 KEGG pathways. The analysis indicated that the active ingredients in Xiangdan injection primarily target several key pathways, including PI3K-Akt, TNF, AGE-RAGE (associated with diabetic complications), IL-17, HIF-1, T cell receptor, JAK-STAT, NOD-like receptor, p53, NF-κB, Toll-like receptor, and Fc epsilon RI signaling pathways. Additionally, they are involved in hepatitis B/C, alcoholic and non-alcoholic fatty liver disease, Th1/Th2/Th17 cell differentiation, and insulin resistance. These findings suggest that Xiangdan injection may modulate cancer-related fatigue, liver injury, and allergic responses through multiple biological mechanisms (Figure 4).

### Molecular docking verification

**FIGURE 5:**
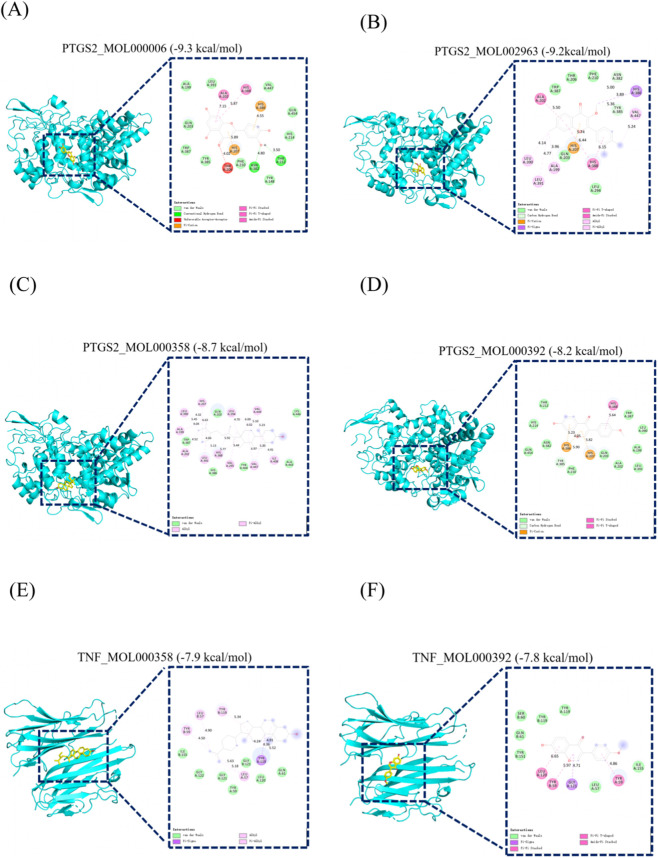
Molecular docking models illustrating the binding of key chemical constituents to potential targets. **(A)** Docking model of PTGS2 in complex with luteolin. **(B)** Docking model of PTGS2 in complex with 4′,5′,7′-trimethyl-3-methoxyflavone. **(C)** Docking model of PTGS2 in complex with beta-sitosterol. **(D)** Docking model of PTGS2 in complex with formononetin. **(E)** Docking model of TNF in complex with beta-sitosterol. **(F)** Docking model of TNF in complex with formononetin.

To validate the predictions from network pharmacology, we conducted molecular docking analyses between key bioactive compounds and their core target proteins. The results indicated favorable binding affinities for all five compounds with their respective target proteins. Notably, PTGS2, TNF, and TP53 showed the strongest binding interactions with several compounds. All binding energies were below −5.6 kcal/mol, with the lowest reaching −9.3 kcal/mol. These findings suggest that these proteins are promising binding targets for the compounds under investigation. The molecular docking results are illustrated in Figure 5.

### The potential mechanisms of the risk of irrational drug use

We conducted an investigation into the mechanisms underlying the risks associated with irrational drug use, utilizing network pharmacology and molecular docking analyses. Luteolin, present in Xiangdan injection, may alleviate cancer-related fatigue by modulating the PI3K/AKT and HIF-1 signaling pathways. These signaling pathways may reduce fatigue by enhancing mitochondrial function. KEGG enrichment analysis indicated that the active components of Xiangdan injection primarily target pathways related to hepatitis B, hepatitis C, and both alcoholic and non-alcoholic fatty liver diseases. This suggests their potential involvement in liver metabolic and inflammatory regulation, which may contribute to hepatic effects under conditions of prolonged use or metabolic saturation. Furthermore, inappropriate solvents may destabilize the pH or osmotic pressure of Xiangdan injection. GO analysis indicated involvement in the “xenobiotic stimulus response,” suggesting the potential for allergic or hypersensitivity reactions to Xiangdan injection.

## Discussion

This study systematically analyzed the main types and potential risks of irrational drug use related to Xiangdan Injection in clinical settings. The clinical audit was supplemented with exploratory network pharmacology and molecular docking analyses to generate testable hypotheses regarding its potential molecular basis, thereby contributing to a more evidence-informed perspective on its clinical use.

### Interpretation of key findings and comparison with literature

Our audit revealed a high rate (62.0%) of inappropriate use, predominantly for unapproved indications such as cancer-related fatigue (CRF) in oncology patients. These findings align with broader pharmacovigilance reports documenting the prevalent off-label use of herbal injections grounded in traditional concepts rather than demonstrated efficacy. The hypothesized biological rationale for such use is partly informed by the known bioactive constituents of Xiangdan Injection. Key compounds identified in our analysis include luteolin, β-sitosterol, and formononetin from *Dalbergia odorifera*, alongside tanshinone IIA and salvianolic acid B from *Salvia miltiorrhiza*. These components possess documented anti-inflammatory, antioxidant, and vasoactive properties. While our network analysis suggests that luteolin in Xiangdan may modulate pathways such as PI3K/AKT associated with fatigue, robust clinical evidence is lacking. This practice may pose undue risks, such as an increased bleeding tendency in susceptible patients, underscoring the critical need for rigorous trials to evaluate this common off-label application. The risk associated with prolonged use (>14 days) was highlighted by a case of asymptomatic elevation of liver enzymes. Although some Xiangdan compounds are reported to have hepatoprotective properties, the potential for hepatotoxicity from other components or metabolic saturation during extended therapy cannot be ruled out. This concern is echoed in pharmacovigilance literature on herbal products. Therefore, our recommendation to limit treatment to ≤14 days per course is prudent and aligns with general safety principles for herbal injections and with specific guideline suggestions. Regular monitoring of liver function is essential for any extended use.

### Mechanisms of solvent-related and pseudoallergic reactions

The use of normal saline as a diluent was associated with dermal reactions in our study. Mechanistically, pH changes in saline can precipitate active ingredients, increasing particulate matter and instability. Recent studies (2022–2024) indicate that pseudoallergic reactions-not IgE-mediated allergies-substantially contribute to immediate hypersensitivity reactions to traditional Chinese medicine injections (Tingqian, 2010; Wang et al., 2020). Such reactions involve direct mast cell activation and degranulation, releasing mediators such as β-hexosaminidase (β-Hex), a sensitive biomarker of these events (Zhang et al., 2025; Ying et al., 2017). This underscores that strict adherence to the recommended 5% glucose diluent is a critical, nonnegotiable safety measure.

### Risk of therapeutic duplication

We identified therapeutic duplication in 7% of cases when Xiangdan was co-administered with other herbal products containing similar blood-activating components. This practice is analogous to dual antiplatelet therapy in Western medicine and may potentiate anticoagulant effects, increasing bleeding risk. A comprehensive medication reconciliation is essential to prevent these hazardous combinations.

### Clinical implications and recommendations for practice

Based on these findings, we propose targeted strategies to enhance hospital medication safety policies. (1) Strict governance of indications: Implement electronic medical record alerts or mandatory field documentation to ensure use only for approved indications. (2) Duration and monitoring protocol: Establish a default maximum therapy duration of 14 days; any extension requires documented re-evaluation and scheduled liver function tests. (3) Solvent safety enforcement: Embed the recommended solvent (5% glucose) in the electronic prescribing system to prevent selection errors. (4) Duplication risk alert: Integrate a pharmacy review system to flag concurrent prescriptions of Xiangdan and other herbs or pharmaceuticals with antiplatelet or anticoagulant properties. (5) Vigilance during infusion: Mandate monitoring of vital signs during the first infusion and ensure readily available emergency management protocols for hypersensitivity reactions.

### Limitations and future perspectives

This study has several limitations. First, the single-center, retrospective design limits generalizability. Although the sample size is informative, validation with larger, multicenter studies is warranted. Second, adverse drug reactions were identified from retrospective medical records; incomplete data may have led to underreporting or misattribution, underestimating their true incidence. Third, and most critically, the network pharmacology and molecular docking results are inherently hypothesis-generating (Priyanka and Jitendra, 2023; Rahman et al., 2024). A key consideration is that the standard bioavailability and drug-likeness (OB/DL) filters applied do not screen for pan-assay interfering compounds (PAINS). Therefore, some of the prioritized bioactive components, while computationally promising, may carry a risk of nonspecific binding or assay interference that could lead to false-positive interpretations in downstream experimental validation. Furthermore, the compound-target associations sourced from public databases may contain propagated errors and biases inherent to their curation process, which could affect the specificity of the constructed network. Their primary value is to prioritize candidate interactions for subsequent *in vitro* and *in vivo* verification. Thus, the value of these *in silico* findings lies primarily in prioritizing candidate interactions for future verification, not in defining established mechanisms.

Notably, although enriched pathways such as PI3K/AKT and HIF-1 participate in broad cellular processes, their relevance to this study was strengthened by a multi-step analytical strategy (Tingting, 2022; Semenza, 2013; Faton and Bing-Hua, 2013). The analysis focused on targets intersecting with the specific clinical conditions under investigation. Core targets within these pathways (e.g., AKT1, TNF) showed high centrality in the condition-specific protein interaction network. Furthermore, molecular docking indicated strong binding between key bioactive compounds (e.g., luteolin) and these core targets. Collectively, this approach proposes a convergent, condition-relevant hypothesis for the bioactivity of Xiangdan injection, rather than merely reflecting nonspecific background signaling. Nevertheless, this mechanistic hypothesis-particularly regarding efficacy for cancer-related fatigue-requires confirmation through rigorously designed prospective clinical trials and functional studies. Despite these limitations, the integrated methodology-combining clinical audit with computational pharmacology-provides a comprehensive framework for evaluating the rational use of herbal injections.

## Conclusion

In summary, clinical risks associated with Xiangdan Injection are predominantly linked to inappropriate use patterns. To mitigate these risks and safeguard patient safety, clinical practice must rigorously adhere to the following core principles: (1) Administration should be strictly limited to approved indications to prevent unwarranted off-label use. (2) Limit treatment duration to a maximum of 14 days per course, with mandatory monitoring for prolonged therapy. (3) Use only the recommended 5% glucose injection as the solvent. (4) Avoid duplication of therapy with other herbal medicines that have similar pharmacological effects. A careful, individualized assessment of risks and potential benefits is essential prior to administration. Future prospective studies and robust clinical trials are warranted to evaluate the efficacy and safety of common off-label uses, including management of cancer-related fatigue.

## Data Availability

The original contributions presented in the study are included in the article/supplementary material, further inquiries can be directed to the corresponding author.
